# Exercise Training in Athletes with Bicuspid Aortic Valve Does Not Result in Increased Dimensions and Impaired Performance of the Left Ventricle

**DOI:** 10.1155/2014/238694

**Published:** 2014-01-30

**Authors:** Laura Stefani, Giorgio Galanti, Gabriele Innocenti, Roberto Mercuri, Nicola Maffulli

**Affiliations:** ^1^Sport Medicine Center, University of Florence, Viale Morgagni 45, Florence, Italy; ^2^Department of Musculoskeletal Disorders, Faculty of Medicine and Surgery, University of Salerno, Salerno, Italy; ^3^William Harvey Institute, Centre for Sports and Exercise, Barts and The London School of Medicine and Dentistry, Queen Mary University of London, UK

## Abstract

*Background.* Bicuspid aortic valve (BAV) is one of the most common congenital heart disease (0.9%–2%) and is frequently found in the athletes and in the general population. BAV can lead to aortic valve dysfunction and to a progressive aortic dilatation. Trained BAV athletes exhibit a progressive enlargement of the left ventricle (LV) compared to athletes with normal aortic valve morphology. The present study investigates the possible relationship between different aortic valve morphology and LV dimensions. *Methods.* In the period from 2000 to 2011, we investigated a total of 292 BAV subjects, divided into three different groups (210 athletes, 59 sedentaries, and 23 ex-athletes). A 2D echocardiogram exam to classify BAV morphology and measure the standard LV systo-diastolic parameters was performed. The study was conducted as a 5-year follow-up echocardiographic longitudinal and as cross-sectional study. *Results.* Typical BAV was more frequent in all three groups (68% athletes, 67% sedentaries, and 63% ex-athletes) than atypical. In BAV athletes, the typical form was found in 51% (107/210) of soccer players, 10% (21/210) of basketball players, 10% track and field athletics (20/210), 8% (17/210) of cyclists, 6% (13/210) swimmers, and 15% (32/210) of rugby players and others sport. Despite a progressive enlargement of the LV (*P* < 0.001) observed during the follow-up study, no statistical differences of the LV morphology and function were evident among the diverse BAV patterns either in sedentary subjects or in athletes. *Conclusion.* In a large population of trained BAV athletes, with different prevalence of typical and atypical BAV type, there is a progressive nonstatistically significant enlargement of the LV. In any case, the dimensions of the LV remained within normal range. The metabolic requirements of the diverse sport examined in the present investigations do not seem to produce any negative impact in BAV athletes

## 1. Introduction 

Bicuspid aortic valve (BAV) is one of the most common congenital heart disease (0.9%–2%) and is frequently found in athletes and in the general population [1]. BAV can lead to aortic stenosis, aortic regurgitation, infective endocarditis, and progressive aortic dilatation [2, 3]. Different spatial orientations of the BAV have been identified [4]. The most frequent BAV subtypes are those with fusion of the right and noncoronary leaflets (R-NC) and those with fusion of right and left coronary leaflets (R-L) [5]. Patients with a BAV with R-NC fusion have a higher risk of aortic stenosis and regurgitation. In addition, the elastic properties of the aorta deteriorate, and the dimensions of the aorta tend to increase [6]. Patients with R-L BAV exhibit a more severe degree of aortic wall degeneration than patients with R-NC BAV, and in addition R-L BAV is associated with coarctation of the aorta (CoA) [7]. Studies in children and adolescents have proven that R-NC BAV subjects show a more rapid progression of aortic valve stenosis and regurgitation [8]. These data suggest that the etiological factors that determine the formation of R-NC and R-L BAVs can also be involved in the occurrence and progression of pathologies associated with each BAV subtype. More recently, in addition to the progressive enlargement of the ascending aorta, a progressive increase of the left ventricle (LV) dimensions has been also demonstrated [9], especially in BAV athletes with mild aortic valve insufficiency. In BAV athletes, the LV dimensions remain within the normal range, although such dimensions are higher compared to subjects with normal tricuspid aortic valve. Currently, it remains unknown whether the different BAV subtypes are associated to different patterns of changes in LV dimensions. The present study aims to verify in a wide population of BAV subjects the prevalence of the different morphologies of bicuspid aortic valve subtypes and the possible association of these subtypes with a more evident enlargement of the LV chamber dimensions.

## 2. Materials and Methods 

### 2.1. Subjects Studied

In the period from 1 January 2000 to 31 December 2011, 292 consecutive subjects with BAV were evaluated at the Sports Medicine and Exercise Centre of the University of Florence. The group included active athletes, ex-athletes, and sedentary subjects.

The active athletes group was composed of subjects who undertook competitive sport, trained regularly, and underwent yearly evaluation at the Sports Medicine and Exercise Centre to obtain eligibility to practice sports. The ex-athletes were subjects who spontaneously decided to stop their training and sport activity for nonhealth related reasons. Sedentary subjects had never practised regular sport activity. The formula weight (kg)/height in (m^2^) was applied to calculate the body mass index (BMI). In all groups, the presence of BAV was investigated by a 2D echocardiographic exam to estimate the prevalence of the different BAV morphologies. Only in the athletes was the possible LV morphology and function modifications associated to a diverse BAV morphology studied. Among the athletes, only the subjects with a complete 5-year followup were investigated. In the other two groups, the presence of BAV and its morphology was assessed.

### 2.2. Echocardiographic Study

All the subjects were investigated by history taking, general physical examination, and 2D echocardiographic exam at rest. The echocardiogram was performed by cardiologists sonographers experts using the echocardiograph My Lab 50 (Esaote, Italy) equipped with a 2.5 MHz transthoracic echocardiographic probe. According to the ACC/AHA guidelines [10], the echocardiographic measurements of the left ventricle (LV) chamber were obtained from the parasternal long-axis view. They included LV end-systolic and end-diastolic diameters (LVEDd, LVESd), interventricular septum (IVS), posterior wall thickness (PW), and the ejection fraction (EF %), calculated according to the formula (LVEDd-LVESd/LVEDd), for which volumes are substituted by diameters. By Doppler analysis, the diastolic variables were measured in the presence of a stable RR interval and in three different but sequential measurements from the four chamber view. They consisted in transmitral flow of E wave and A wave peak velocities, isovolumetric relaxation and deceleration times, and E/A ratio. The degree of severity of the insufficiency, described as the extent of the regurgitant jet on a 0 to 4+ scale, was assessed using the colour-flow mapping method from the four-chamber view, according to the ACC/AHA guidelines [11]. The index of left ventricular mass (LVMI) and left ventricular hypertrophy (LVH) was calculated according to the equation by Devereux [12]. Aortic valve morphology was examined in the parasternal long- and short-axis views. The diagnosis of BAV was confirmed when two cusps and two commissures were clearly identified in systole and diastole in the short-axis view. The aortic dimensions were measured in 2-dimensional parasternal long axis views at the end of diastole in the views at four levels: aortic annulus (Aoan), Valsalva sinuses (Aosv), sinotubular junction (Aostj), and proximal ascending aorta (PAA), measured 1 cm from the stj [13]. Every BAV type was immediately classified according to the presence or absence of a fibrous raphe of one of the two cusps.

In the phenotype “with raphe” three variants of BAV have been identified: the first and most common is characterized by fusion of right and left coronary cusp (R-L), the second presents right and noncoronary fusion (R-NC), and the third, less common variant, presents left and noncoronary fusion (L-NC).

In the phenotype “no raphe,” only two cusps with free margin and commissures are present. The orientation of the commissures and the free margin [14] allows to distinguish anteroposterior (AP) and a lateral (LA) variants.

The evaluation of the aorta was completed measuring the ascending aortic tract at four different levels (aortic annulus (Aoan), sinuses of Valsalva (Aosv), joint sino-tubular (Aostj), proximal ascending aorta (PAA), measured at 1 cm from the joint sino-tubular).

The presence of aortic stenosis (AS) was assessed over the window apical 4-chamber with continuous-wave Doppler. The flow velocity of transvalvular aortic of 2.5 m/sec (corresponding to the maximum gradient of 25 mmHg) was compatible with the presence of stenosis.

Echocardiography also included the evaluation of other possible anatomical or hemodynamic abnormalities, such as the presence of other valvular regurgitation, mitral and/or tricuspid, the calculation of the pulmonary pressure, the presence of CoA, interventricular or interatrial septal defects, or a patent foramen ovale.

## 3. Statistical Analysis

The results are reported as mean ± standard deviation. The differences in the values of the LV among athletes with different morphologies of BAV were evaluated with multivariate analysis using ANOVA. *P* values < 0.05 were considered statistically significant.

## 4. Results

### 4.1. General Data

**Figure 1:**
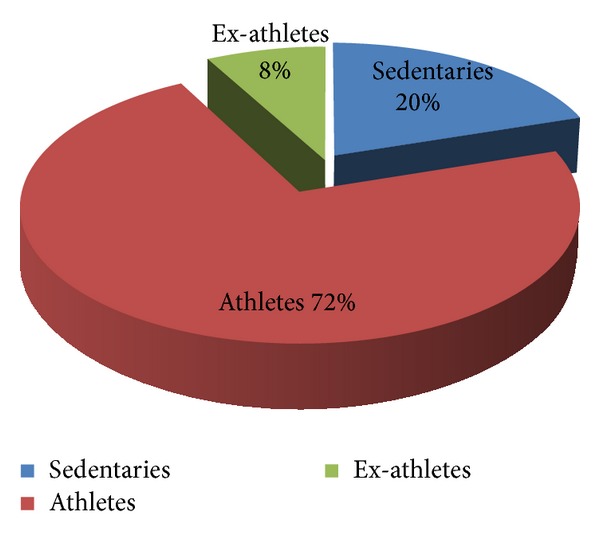
Composition of the whole population investigated (athletes, ex-athletes, and sedentaries).

The BAV population studied (Figure 1) was composed of 292 subjects, including 210 athletes (72% of the population), 23 ex-athletes (8%), and 59 sedentary subjects (20%). The athletes and the sedentary subjects were followed at yearly intervals with echocardiography for at least 5 years. Within all three groups, the morphology of BAV “with raphe” has been more represented than the BAV “without raphe.” BAV “with raphe” was found, respectively, in the 68% (143) of athletes, 65% (16) of the ex-athletes, and 63% (37) of the sedentary subjects. The presence of other associated minor cardiac abnormalities such as mitral valve prolapse (MVP) or patent foramen ovalis (PFO) in all three populations was very low. Only 9/56 of the sedentary subjects however showed a progressive valve dysfunction or dilatation of the ascending aorta and underwent cardiac surgery.

### 4.2. Athletes Group

The BAV athletes were from different types of sports: 51% football, 10% basketball, 10% track and field athletics, 8% cycling, 6% swimming, and the remaining 15% practiced other sports such as rugby, fencing, martial arts, volleyball, tennis, horse-riding, triathlon, diving, dancing, and boxing. All the athletes were competitive athletes and trained at least three times a week for 10 months a year, and, in the competitive season, competed at least once a week. The most common variant found at echocardiography was the BAV “with raphe” with the R-L morphology subtype. It was the most represented in both athletes (70%) and ex-athletes (73%). The R-NC type had a prevalence of 29% in athletes and 27% in the ex-athletes. In the variant bicuspid aortic valve “without raphe,” the AP type has a prevalence of 87% in athletes and 93% in the ex-athletes. The LA type had a prevalence of 13% in athletes and 7% in the ex-athletes. None of the athletes underwent surgery during the study period.

**Figure 2:**
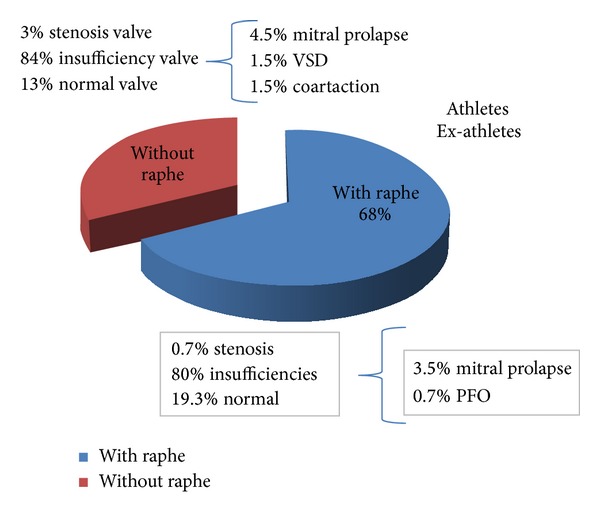
Presence of BAV morphologies and associated cardiac patterns in the whole athletes BAV group.

At colour Doppler exam, the most prevalent aortic valve dysfunction found in athletes was valve insufficiency. It was present in 84% of the whole athletes BAV group (athletes and ex-athletes) without raphe, and in 80% of the BAV athletes with raphe. The degree of aortic valve insufficiency was estimated by the colour Doppler analysis and was always mild. On the contrary, aortic valve stenosis was found in the 3% of the group “without raphe,” and in the 0.7% of the group of BAV athletes with raphe. In the rest, the aortic valve was functioning normally (13% in the subjects without raphe, and 19.3% in the group with raphe). In the same group, other minor congenital cardiac abnormalities were found. For example, a mitral valve prolapse (MVP) was found in the 3.5% of the BAV group with raphe, and in the 4.5% of the no raphe group. A patent foramen ovalis (PFO) was found in 0.7% of the BAV group with raphe. A ventricle septal defect (VSD) was found only in the group of BAV athletes without raphe, as well as the coartaction of aorta in the 1.5% of the same subtype of the athletes group (Figure 2).

### 4.3. Sedentary Group

**Figure 3:**
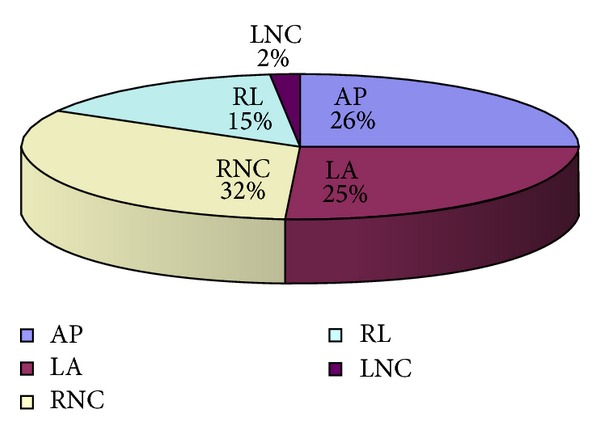
Presence of the BAV morphologies in sedentaries. AP: anteroposterior; LA: lateral; RL: right left; RNC: right non coronary; LNC: left noncoronary.

The sedentary group was composed of 59 subjects, of which 41 had a complete 5-year followup and whose prevalence of the BAV morphology is shown in Figure 3.

The behaviour of the 4 diameters of the aortic tract, with respect to the diverse morphological pattern, showed in sedentaries dimensions higher than the normal subjects with a major enlargement of the BAV LNC variant (Table 1).

**Figure 4:**
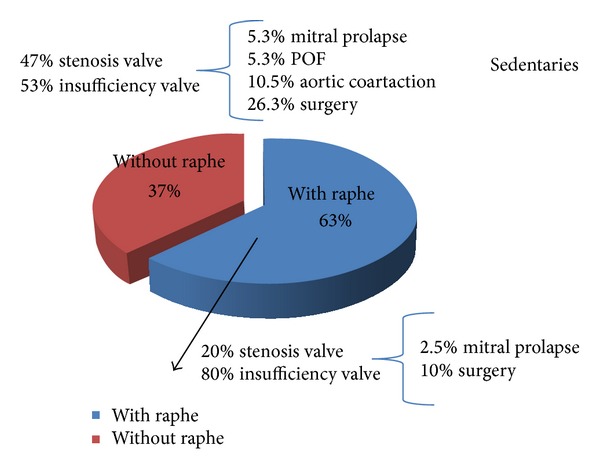
Presence of BAV morphologies and associated cardiac patterns in sedentaries BAV subjects. LV chamber echoparameters of athletes and sedentary subjects with different BAV morphologies.

The most common BAV morphology was R-L with raphe, in 62% of them. The R-NC was found in 35%. The raphe L-NC type had a prevalence of 1%. In the BAV morphology “without raphe,” the AP subtype was found in 68%, and the L-A variant in 37%. The most frequent aortic valve dysfunction was aortic insufficiency, but also a mild aortic stenosis was found only in 7/59 (11%) of the whole group investigated, either “with” or “without” raphe. Some other cardiac associated congenital conditions such as PFO (5.3%), MVP (5.3%), and CoA (10.5%) were also found (Figure 4). Only 26% of the BAV subjects “with raphe” and the 10% of those “without raphe” underwent surgery for the valve dysfunction or progressive aortic dilatation.

The main echocardiographic data of the different types of BAV in athletes are shown in Table 2, with no significant differences between them at the onset of the study. On the contrary, significant differences were found in the LV echocardiography parameters in the group of athletes during the 5 years of the study (Table 3), with a progressive not statistically significant enlargement of the LV chamber for all the BAV groups.

**Figure 5:**
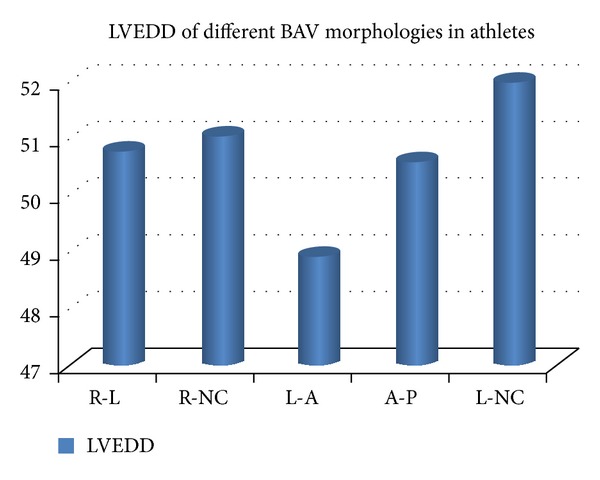
LVEDD dimensions of the BAV morphology after 5 years of followup. LVVD: left ventricle diastolic diameter; R-L: 50.80 ± 4.3 mm; R-NC: 51.04 ± 4.0 mm; LA: 48.92 ± 3.5 mm; AP: 50.69 ± 3.3; L-NC: 52.5 ± 2.5 mm; P: NS for all.

From the Doppler analysis, however, the aortic flow velocity and the aortic gradient remained normal. At the end of the 5 years of the study, the LVDD mean values diameters of every subtype of the BAV morphologies did not show, however, any significant difference (Figure 5).

Among the sedentary subjects, 41 had a complete 5-year followup. They showed a progressive not significant decrease of the values during the observation period for all the LV chamber parameters (Table 4) (LVEDd of R-L BAV type: 48.80 ± 7.3 mm; R-NC: 49.40 ± 9.51 mm; LA: 46.28 ± 7.5 mm; AP: 49.68 ± 8.62; L-NC: 50.5 ± 2 mm; P: NS for all).

On the contrary, significant differences were found comparing the BAV sedentary group with the athletes with respect to all the echocardiography parameters: PW, IVS, RV, and left atrium, aortic flow velocity, CMI, LV EDD and LVESD, and EF (*P* < 0.05). (0.001 < *P* < 0.05). All the values were, however, within the normal range for both groups.

The ex-athletes group was composed of 21 subjects, aged 38.74 ± 12.42, not regularly submitted to a 5-year followup, in consequence of their sports activity discontinuity. The echocardiographyc parameters were within the normal range in this observational period (EF (%) 67.48 ± 4.26; BSA in (m^2^): 1.79 ± 4.26; IVS (mm) 9.78 ± 0.3; PW (mm) 10.04 ± 0.67; LVEDd (mm) 55.01 ± 4.52; LVESd (mm) 36.17 ± 2.59 CMI in (g/m^2^) 109.75 ± 12.78; Aoan (mm) 36.17 ± 5.42; Aosv (mm) 40.52 ± 5.40; Aostj (mm) 40.83 ± 6.94; PAA (mm) 39.39 ± 7.67; E wave (msec) 66.26 ± 14.02; DTc (ms) 77.17 ± 14.61; IVRT (ms) 77.17 ± 14.61; RV (mm) 25.42 ± 2.68). The echocardiographyc feature among the different BAV subtypes, without any significant difference, is reported in Table 5.

**Figure 6:**
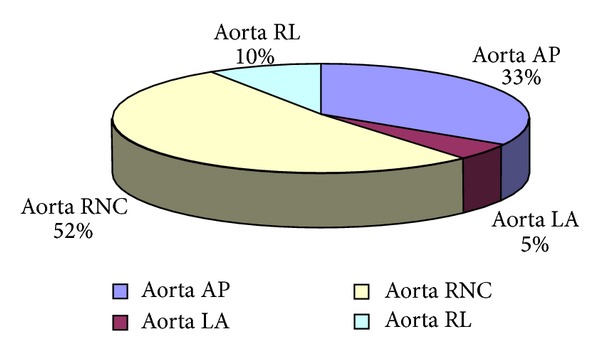
Presence of different BAV morphological patterns in ex-athletes group. AP: anteroposterior; LA: lateral; RL: right left; RNC: right noncoronary: LNC: left noncoronary.

The different morphologies of BAV represented were composed in ex-athletes group of 10% of RL BAV aorta; 33% of AP BAV aorta; 52% of RNC BAV aorta; and 5% of LA BAV aorta (Figure 6).

As expected, the BAV dimensions measured at 4 different levels show a progressive enlargement during the 5-year followup period, with a slightly greater increase in athletes, with no differences between athletes and sedentary subjects. There was a progressive increase at each measured aortic level (Aoan: 0.78 mm/year; Aosv: 0.61 mm/year; Aostj: 0.81 mm/year; PAA: 0.98 mm/year). These values are comparable to those found in previous studies conducted on nonathletic BAV populations [15].

There was no statistical difference in BAV dimensions in the three groups studies. The athletes and the sedentary subject showed significant differences in LV dimensions, although all the values remained within the normal range. The ex-athletes showed higher dimension than the other two groups, but we point out that we do not have longitudinal data on this group.

## 5. Discussion 

BAV is a common congenital condition, more prevalent in males. In athletes, competitive sports activity is normally allowed if the valve dysfunction or the other complications associated are not severe [9, 16]. Some aspects remain unsolved, especially in athletes who train regularly. For example, some changes of the LV have been described [17, 18]. Although they may be considered secondary to the flow dynamics component and to the entity of the aortic valve regurgitation, if present, recent studies have shown that possible structural abnormalities occur at the cellular level, independent of the hemodynamic impairment of BAV subjects [19, 20]. Following this line of thought, LV dimensions have been studied especially in athletes, and a progressive enlargement, however within the normal range, has been recently demonstrated [9]. Using a dedicated software to study the deformation parameters of the LV, normal LV function has been however confirmed. Nevertheless, some other aspects remain unsolved, especially if a large cohort of BAV trained athletes with different BAV types is considered. The diverse BAV types can be associated to a possible more rapid valve dysfunction, and, therefore, possible impairment of the LV.

The present longitudinal study, involving a wide group of BAV subjects with a different prevalence of the BAV morphological variants, investigates the possible relationship of the BAV types with morphological and functional aspects of the LV, thus producing evidence that a given BAV pattern can play a role in predicting the evolution of the morphology and function of the LV.

In the present investigation, the prevalence of the various types of BAV is in agreement with what was previously reported. Particularly in athletes, the prevalence of BAV and the various subtypes are comparable to what was found in the general population, with a predominance of the variant with raphe fusion RL. Also, the most common valve dysfunction pattern associated is valve stenosis, and the prevalence of cardiovascular comorbidity is otherwise rare. Coarctaction of the aorta is, in the present population, rare, with a prevalence of only 1.5%. This is in contrast with the body of available literature where CoA can be present in up to 40% of BAV subjects. Probably, this reflects the fact that the present study was conducted in athletes in whom CoA is a contraindication to the attainment of fitness to practice competitive sports.

In addition, the follow-up echocardiographic data confirm that the LV dimension of BAV athletes tends to be near the upper limits of normality, although slightly increasing during the 5-year followup. On the contrary in BAV sedentaries the LV dimensions remain substantially unchanged if compared to the athletes. In consideration of the fact that all the dimensions are within the normal range for both, the apparent opposite behaviour of the LV chamber of the two different groups is however reasonably interpreted as a physiological heart remodelling influenced by regular physical training. Of note is that the sedentary group also shows a high prevalence (16%) of surgery from the dysfunction of the aortic valve.

Being a carrier of a particular morphology of BAV, according to the results in the present study, cannot predict future development of pathological valve dysfunction.

The mild enlargement of the left ventricle seems to be related to the long term sport training, and therefore compatible with the general feature of athlete's heart. In agreement with the current literature, the ascending aorta is normally involved in progressive, not necessarily pathological, enlargement.

In conclusion, the presence of BAV does not therefore represent an additional risk factor in terms of morphology and left ventricular function, at least in the presence of mild valve dysfunction. This aspect seems to be confirmed also in the presence of those subtypes of valve morphologies well noted for their high risk of progression to valve dysfunction.

From the results obtained in the large group investigated, sport activity does not play any additional role in cardiac morphology and function of trained BAV athletes. Further studies will be necessary to investigate the possible protective role of regular sport participation in this population. A large cohort of subjects, from different types of sports, will be necessary to verify possible differences among the diverse sports practised.

## 6. Limitations and Conclusions

The study has been conducted in a wide population of athletes, but the sedentary group was restricted to relatively few subjects. A wider population will be needed to clarify the possible negative impact of specific sports. Additional information could be also obtained from the application of the deformation parameters to better investigate LV performance. Particularly in this context, evaluation by the 3D echo investigation could play a role. In addition, despite the follow-up study being anyway limited to a 5-year exploration and therefore restricted, it is reasonable to propose an echocardiographic exam at least once a year for all the BAV athletes.

## Figures and Tables

**Table 1:** Dimensions of the aortic tract in BAV sedentaries with diverse morphological patterns.

Sedentary	Aoan (mm)	Aosv (mm)	Aostj (mm)	PAA (mm)
BAV AP	34.66 ± 4.75	37.46 ± 4.91	37.53 ± 5.51	36.6 ± 6.28
BAV LA	33.6 ± 4.36	36.73 ± 4.76	36.6 ± 6.49	35.33 ± 6.78
BAV RL	38.22 ± 6.63	42.22 ± 6.40	40.55 ± 7.54	41.55 ± 7.49
BAV RNC	33.26 ± 4.86	36.63 ± 4.95	36.47 ± 4.89	36.21 ± 5.12
BAV LNC	38	45	43	43

BAV: bicuspid aortic valve; AP: anteroposterior; LA: lateral; RL: right left; RNC: right noncoronary; LNC: left noncoronary.

**Table 2:** Echocardiographic features of the athletes with different types of bicuspid aortic valve.

	R-L = 101	R-NC = 41	L-NC = 2	A-P = 59	L-A = 8	*P *
Age	29.11 ± 12.12	29.36 ± 12.03	28.83 ± 12.37	29.24 ± 12.05	28.97 ± 12.00	NS
EF (%)	60 ± 18	59 ± 18	62 ± 10	59 ± 18	60 ± 18	NS
BSA in (m^2^)	1.80 ± 0.2	1.81 ± 0.2	1.79 ± 0.29	1.81 ± 0.2	1.81 ± 0.2	NS
IVS (mm)	9.42 ± 1.5	9.47 ± 1.4	9.34 ± 1.32	9.46 ± 1.4	9.50 ± 1.3	NS
PW (mm)	9.18 ± 1.5	9.23 ± 1.3	9.10 ± 1.32	9.23 ± 1.3	9.27 ± 1.2	NS
LVEDd (mm)	50.12 ± 7.4	50.42 ± 6.6	49.97 ± 4.44	50.39 ± 6.6	50.61 ± 5.5	NS
LVESd (mm)	31.52 ± 5.1	31.70 ± 4.7	31.88 ± 2.91	31.71 ± 4.7	31.86 ± 4.2	NS
CMI in (g/m^2^)	111.66 ± 23.2	111.83 ± 23.4	110.98 ± 19.9	111.58 ± 23.3	111.04 ± 21.9	NS
Aoan (mm)	31.15 ± 4.2	31.26 ± 4.2	30.52 ± 5.00	31.19 ± 4.2	31.10 ± 4.2	NS
Aosv (mm)	33.83 ± 4.6	33.96 ± 4.5	33.26 ± 6.08	33.90 ± 4.5	33.85 ± 4.5	NS
Aostj (mm)	33.00 ± 4.7	33.10 ± 4.6	32.73 ± 6.01	33.03 ± 4.6	33.96 ± 4.7	NS
PAA (mm)	32.30 ± 4.92	32.45 ± 4.8	31.36 ± 6.58	32.35 ± 4.88	32.25 ± 4.8	NS
E wave (msec)	80.60 ± 23.4	78.30 ± 22.5	76.4 ± 21.4	81.32 ± 19.3	77.22 ± 24.5	NS
A wave (msec)	43.87 ± 14.3	50.18 ± 2	55.23 ± 25	45.90 ± 22.3	44.80 ± 25.3	NS
DTc (ms)	176.71 ± 46.8	180.50 ± 23	179.80 ± 34.5	168.80 ± 27.3	184.46 ± 25.6	NS
IVRT (ms)	73.28 ± 13	70.52 ± 15	68.45 ± 12	74.52 ± 15	82.67 ± 16	NS
RV (mm)	25.34 ± 12	24.23 ± 10	23.25 ± 14	25.23 ± 15	26.24 ± 15	NS

EF: ejection fraction; BSA: body surface area; IVS: interventricular septum; PW: posterior wall; LVEDd: left ventricle diastolic diameter; LVESd: left ventricle systolic diameter; CMI: cardiac mass index; Aoan: aortic annulus; Aosv: aortic dimension at Valsalva sinuses; Aostj: aortic dimension at sinotubular junction; PAA: dimension of proximal ascending aorta; DTc: deceleration time; IVRT: isovolumic relaxation time; RV: right ventricle.

**Table 3:** BAV athletes: details of echocardiographic parameters during the 5-year followup.

	Year 1	Year 2	Year 3	Year 4	Year 5	*P *
AGE	19 ± 8.8	20 ± 8.5	21 ± 8.9	22 ± 8.8	23 ± 9.0	
IVS (mm)	8.97 ± 1.1	9.30 ± 1	9.43 ± 1.02	9.82 ± 0.7	9.89 ± 0.7	<0.01
PW (mm)	8.57 ± 1.0	9.01 ± 0.9	9.12 ± 0.9	9.53 ± 0.8	9.57 ± 0.8	<0.01
LVEDd (mm)	49.66 ± 4	49.51 ± 5	50.27 ± 4.2	51.47 ± 3.5	52.01 ± 3.9	<0.01
LVESd (mm)	30.61 ± 3.5	30.93 ± 3.8	31.37 ± 2.9	32.66 ± 3.2	32.62 ± 2.9	<0.01
CMI in (g/m^2^)	103.11 ± 21.0	106.78 ± 23.6	107.79 ± 22.3	115.11 ± 20.8	117.40 ± 20.6	<0.01
EF (%)	67.22 ± 4.1	67 ± 3.4	67 ± 2.5	65.44 ± 3.0	65.59 ± 3.3	0.074
Aortic vel. (m/sec)	1.64 ± 0.3	1.64 ± 0.3	1.67 ± 0.3	1.65 ± 0.3	1.63 ± 0.3	NS
Ao valve gradient (mmHg)	11.44 ± 5.6	11.24 ± 4.6	11.72 ± 5.2	11.38 ± 4.9	11.15 ± 5.1	NS

EF: ejection fraction; IVS: interventricular septum; PW: posterior wall; LVDd: left ventricle diastolic diameter; LVSd: left ventricle systolic diameter; CMI: cardiac mass index; Ao valve gradient: aortic valve gradient.

**Table 4:** BAV sedentary: details of echocardiographic parameters during the 5-year followup.

	Year 1	Year 2	Year 3	Year 4	Year 5	*P *
AGE	30 ± 8.1	31 ± 7.5	32 ± 9.2	33 ± 9.5	34 ± 8.3	
IVS (mm)	9.63 ± 2.38	9.39 ± 2.69	9.19 ± 2.87	8.87 ± 3.09	8.71 ± 3.15	NS
PW (mm)	9.47 ± 2.36	9.25 ± 2.66	9.07 ± 2.84	8.76 ± 3.06	8.60 ± 3.12	NS
LVEDd (mm)	49.41 ± 12.24	48.08 ± 13.77	47.15 ± 14.70	45.38 ± 15.74	44.58 ± 16.06	NS
LDDS (mm)	28 ± 7.99	30.46 ± 8.90	29.84 ± 9.46	28.67 ± 9.91	28.16 ± 10.11	NS
CMI in (g/m^2^)	95.44 ± 14.52	97.85 ± 15.42	96.55 ± 16.68	98.67 ± 14.68	95.97 ± 16.85	NS
EF (%)	65.22 ± 4.1	64 ± 3.4	66.54 ± 2.5	65.65 ± 3.0	63.85 ± 3.3	NS
Aortic vel. (m/sec)	1.35 ± 0.4	1.40 ± 0.2	1.37 ± 0.3	1.34 ± 0.3	1.38 ± 0.3	NS
Ao valve gradient (mmHg)	7.29 ± 4.23	7.84 ± 5.12	7.51 ± 4.92	7.18 ± 4.68	7.62 ± 5.03	NS

EF: ejection fraction; IVS: interventricular septum; PW: posterior wall; LVDd: left ventricle diastolic diameter; LVSd: left ventricle systolic diameter; CMI: cardiac mass index; Ao valve gradient: aortic valve gradient.

**Table 5:** Echocardiographic features of the ex-athletes with different types of bicuspid aortic valve.

	R-L = 11	R-NC = 4	A-P = 7	L-A = 1	*P *
Age	37.45 ± 12.79	36.85 ± 11.63	38.05 ± 12.75	34.00 ± 0	NS
EF (%)	67 ± 3	66 ± 5	64 ± 7	66	NS
BSA in (m^2^)	1.78 ± 0.2	1.82 ± 0.2	1.80 ± 0.2	1.80 ± 0	NS
IVS (mm)	10.13 ± 0.97	9.25 ± 0.5	9.64 ± 0.95	9.00	NS
PW (mm)	10.32 ± 0.60	9.50 ± 0.58	10.07 ± 0.60	9.00	NS
LVEDd (mm)	56.27 ± 4.54	56.75 ± 2.22	55.71 ± 5.62	50.00	NS
LVESd (mm)	36.36 ± 3.35	35.75 ± 0.96	36.71 ± 2.82	32.00	NS
CMI in (g/m^2^)	110.54 ± 20.6	107.64 ± 20.7	109.78 ± 20.3	105	NS
Aoan (mm)	36.45 ± 5.22	37.00 ± 5.16	36.15 ± 6.47	30.00	NS
Aosv (mm)	42.09 ± 5.00	39.75 ± 5.86	39.42 ± 5.89	34.00	NS
Aostj (mm)	33.00 ± 4.7	39.00 ± 8.20	41.57 ± 8.28	29.00	NS
PAA (mm)	32.30 ± 4.92	38.50 ± 5.8	40.14 ± 9.20	26.00	NS
Ewave (msec)	70.36 ± 13.22	54.00 ± 5.41	65.85 ± 16.60	73.00	NS
Awave (msec)	47.90 ± 10.12	37.75 ± 4.5	44.86 ± 12.52	52.00	NS
DTc (ms)	188.45 ± 32.33	200.00 ± 35.60	180.14 ± 23.73	171.00	NS
IVRT (ms)	73.73 ± 14.73	89.50 ± 1.92	77.71 ± 16.02	62.00	NS
RV (mm)	26.12 ± 11.23	25.73 ± 10.25	25.85 ± 13.65	25.00	NS

EF: ejection fraction; BSA: body surface area; IVS: interventricular septum; PW: posterior wall; LVEDd: left ventricle diastolic diameter; LVESd: left ventricle systolic diameter; CMI: cardiac mass index; Aoan: aortic annulus; Aosv: aortic dimension at Valsalva's sinuses; Aostj: aortic dimension at sinotubular junction; PAA: dimension of proximal ascending aorta; DTc: deceleration time; IVRT: isovolumic relaxation time; RV: right ventricle.
